# Contribution of *IL6* −174 G>C and *IL1B* +3954 C>T polymorphisms to congenital infection with *Toxoplasma gondii*

**DOI:** 10.1007/s10096-015-2481-z

**Published:** 2015-09-18

**Authors:** W. Wujcicka, Z. Gaj, J. Wilczyński, D. Nowakowska

**Affiliations:** Scientific Laboratory of the Center of Medical Laboratory Diagnostics, Polish Mother’s Memorial Hospital—Research Institute, 281/289 Rzgowska Street, Lodz, 93-338 Poland; Department of Perinatology and Gynecology, Polish Mother’s Memorial Hospital—Research Institute, Lodz, Poland

## Abstract

The purpose of this investigation was the determination of the distribution of genotypes and alleles, residing within interleukin 6 (*IL6*) and interleukin 1 (*IL1*) polymorphisms, among fetuses and neonates, congenitally infected with *Toxoplasma gondii*, and among uninfected control cases. The study included 22 fetuses and newborns infected with *T. gondii* and 49 control cases. Screening for IgG and IgM antibodies against the parasite and IgG avidity was performed by enzyme-linked fluorescent assay (ELFA) tests. Quantitation of *T. gondii* DNA in amniotic fluids was assayed by the real-time Q PCR technique for the parasitic *B1* gene. Genotypes at *IL6* and *IL1* single nucleotide polymorphisms (SNPs) were determined by a self-designed, nested polymerase chain reaction-restriction fragment length polymorphism (PCR-RFLP) assay. Representative genotypes at the studied loci were confirmed by sequencing. All the genotypes were estimated for Hardy–Weinberg equilibrium and *IL1* genotypes were tested for linkage disequilibrium. Genotypes and haplotypes at the studied SNPs were investigated for their possible association with the occurrence of congenital *T. gondii* infection, using a logistic regression model. GC heterozygotes at the *IL6* −174 G>C SNP were significantly associated with toxoplasmosis and increased the risk of *T. gondii* infection [odds ratio (OR) 4.24, 95 % confidence interval (CI) 1.24–14.50 in the codominant model, *p* ≤ 0.050]. In case of *IL1* SNPs, similar prevalence rates were observed between *T. gondii*-infected and -uninfected offspring. Regarding allelic variability, the C alleles at both *IL6* and *IL1B* SNPs were significantly more frequent in the infected than in the uninfected cases (*p* ≤ 0.050). It is concluded that *IL6* −174 G>C and *IL1B* +3954 C>T SNPs might be involved in the development of congenital *T. gondii* infection.

## Introduction

*Toxoplasma gondii* is one of the most common intrauterine infections worldwide and the major cause of perinatal morbidity and mortality [1–4]. Congenital infections with *T. gondii* are diagnosed in about 0.07–2.9 % of live newborns, leading to asymptomatic as well as symptomatic disease, sometimes with fatal course [1, 5, 6].

Considering the immune response against *T. gondii*, several studies have shown the role of proinflammatory cytokines, including interleukin (IL) 6 and IL1 [7–9]. Among women infected with *T. gondii*, IL6 expression was estimated to be twice as high compared to the control cases [10]. In another study, the development of toxoplasmosis-related ocular lesions among *T. gondii*-infected patients was reported to be associated with high IL1 and TNF-α levels [11]. In turn, the mice with more severe inflammation in the retina and vitreous humor, related to ocular toxoplasmosis, had a reduced expression of ocular *IL1A* and an increased production of TNFA mRNA as compared to WT mice [12]. In an in vitro study, the human monocytic wild-type MonoMac6 cells, infected with *T. gondii*, showed significantly higher levels of IL-1β as compared to non-infected cells [13]. Considering a possible participation of the genetic alterations, located at *IL1* and *IL6* molecule encoding genes, the −174 G>C single nucleotide polymorphism (SNP) from the *IL6* gene was reported to be correlated with toxoplasmic retinochoroiditis (TR) [14]. The prevalence rates of genotypes and alleles at the *IL6* −174 G>C SNP differed significantly between the patients with TR and the healthy blood donors with positive serology for *T. gondii* infection and without retinal symptoms of the previous disease [14]. In turn, another study reported no correlation of either *IL1A* −889 C>T or *IL1B* C>T SNPs with the occurrence of TR [15]. However, distinct differences in the distribution of genotypes and alleles at the *IL1A* SNP were observed between patients with and without recurrent episodes of the disease [15]. So far, no study has been performed to seek for a possible involvement of *IL1* and *IL6* SNPs in the development of congenital infection with *T. gondii*.

In the reported study, we aimed to analyze and describe a possible influence of the polymorphisms located at the *IL1A*, *IL1B*, and *IL6* genes on the occurrence of congenital *T. gondii* infection in fetuses and neonates. The haplotype prevalence rates were also estimated for *IL1* SNPs. A multiple-SNP analysis was performed to estimate the assumed simultaneous influence of *IL1* and *IL6* SNPs on the occurrence of the disease.

## Materials and methods

The study included 22 fetuses congenitally infected with *T. gondii* and 49 uninfected control cases, from which samples were retrospectively (16 infected cases and 25 controls) as well as prospectively (six infected cases and 24 controls) collected. The fetal and neonatal specimens were obtained from the offspring of pregnant women treated at the Department of Fetal-Maternal Medicine and Gynecology at the Polish Mother’s Memorial Hospital—Research Institute in Lodz, between the years 2000 and 2014. Clinical samples, selected for genetic studies, consisted of fetal amniotic fluids, obtained via amniocentesis from pregnant women, and/or cerebrospinal fluids (six samples), as well as umbilical cord blood specimens (two samples). The intrauterine infections with *T. gondii* were determined from maternal serological features of the recent infection and by the clinical picture, including flu-like symptoms, as well as by fetal and neonatal ultrasound markers associated with toxoplasmosis. The congenital infections with *T. gondii* were confirmed by the presence of parasitic DNA in fetal and neonatal body fluids. The research was approved by the Research Ethics Committee at the Polish Mother’s Memorial Hospital—Research Institute. All the samples, previously collected for diagnostic purposes, are anonymized in this report. Informed consent forms were signed by all the enrolled participants (pregnant women).

### Serological tests

Blood specimens were collected from the pregnant women by venipuncture on their first visit to the hospital. Serum samples were obtained by centrifugation and then stored at 4 °C until analysis. Serological tests were performed at the hospital’s Department of Clinical Microbiology.

Screening for *T. gondii* IgG antibodies was performed with the enzyme-linked fluorescent assay (ELFA) VIDAS TOXO IgG II (bioMérieux). The IgM antibodies were detected with the ELFA assay VIDAS TOXO IgM (bioMérieux). *T. gondii* IgG avidity was assayed by an ELFA assay VIDAS TOXO IgG AVIDITY (bioMérieux). The pregnant women were diagnosed as recently infected with *T. gondii* in case of seroconversion during pregnancy or were suspected to be infected in case of the infection-related serology including IgM seropositivity and low IgG avidity index. Fresh intrauterine *T. gondii* infections, possible in the studied pregnant women, were confirmed by real-time Q PCR assays for the parasitic *B1* gene fragments, performed on body fluid specimens of their offspring.

### DNA extraction

Fetal genomic DNA was extracted from 5-mL amniotic fluid volumes or 3-mL cerebrospinal fluid volumes, and neonatal DNA was extracted from 200 μL of umbilical cord blood specimens, using a High Pure PCR Template Preparation Kit (Roche™, Mannheim, Germany). The obtained DNA was diluted in 100 μL of elution buffer and stored at −20 °C until further molecular analyses.

### Detection and quantification of *T. gondii* loads

The amount of *T. gondii* DNA was quantified by a real-time (RT) Q PCR assay for the detection of parasitic *B1* gene fragment of 83 bps in length. Forward and reverse primers and TaqMan MGB probe sequences were as follows: 5′-CAAGCAGCGTATTGTCGAGTAGAT-3′, 5′-GCGTCTCTTTCATTCCCACATTTT-3′, and 5′-6-FAM- CAGAAAGGAACTGCATCCGTT-NFQ-3′, respectively [16]. The reactions were prepared in final 25-μL volumes, as described previously [17].

### Determination of SNPs located within *IL1* and *IL6* genes

**Fig. 1:**
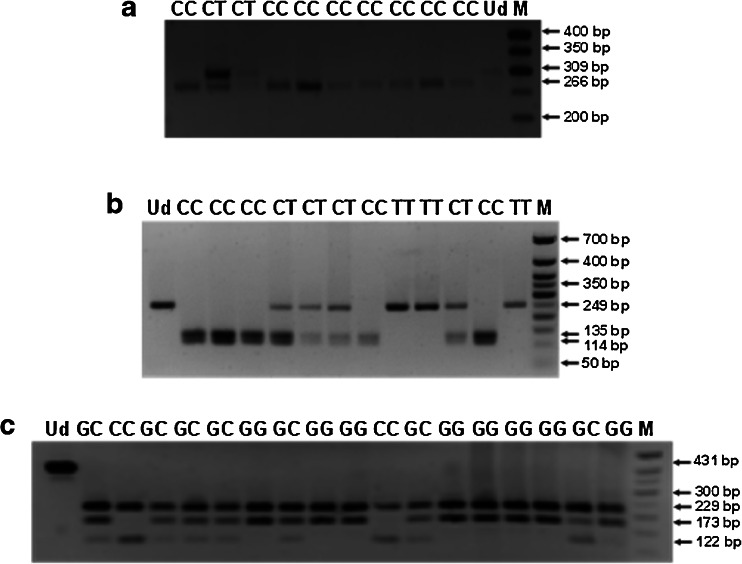
Agarose gel electrophoresis of polymerase chain reaction-restriction fragment length polymorphism (PCR-RFLP) products for distinct genotypes at *IL1A* −889 C>T single nucleotide polymorphism (SNP) (**a**), *IL1B* +3954 C>T SNP (**b**), and *IL6* −174 G>C SNP (**c**). DNA fragments, obtained by the restriction analyses with NcoI (**a**), TaqI (**b**), and Hsp92II (**c**) endonucleases, were resolved in 2 % agarose gels, stained with ethidium bromide. The numbers on the right indicate the size of separated DNA fragments. *M* 50-bp DNA marker; *Ud* undigested PCR product; *CC*, *CT*, *TT*, *GG*, *GC*, *CC* genotypes at the studied *IL* polymorphisms

**Fig. 2:**
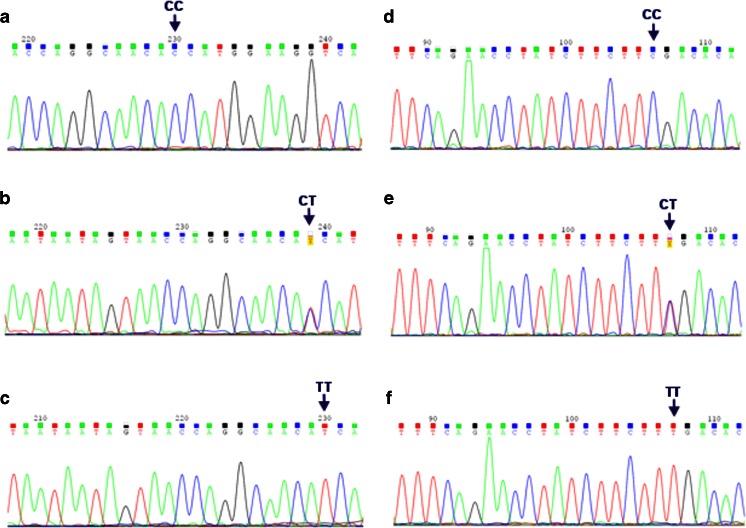
DNA sequences, containing *IL1A* −889 C>T SNP (**a**, **b**, **c**) and *IL1B* +3954 C>T SNP (**d**, **e**, **f**). DNA forward strands were sequenced for all the analyzed SNPs. *CC*, *CT*, *TT* genotypes at the described *IL1* SNP

**Fig. 3:**
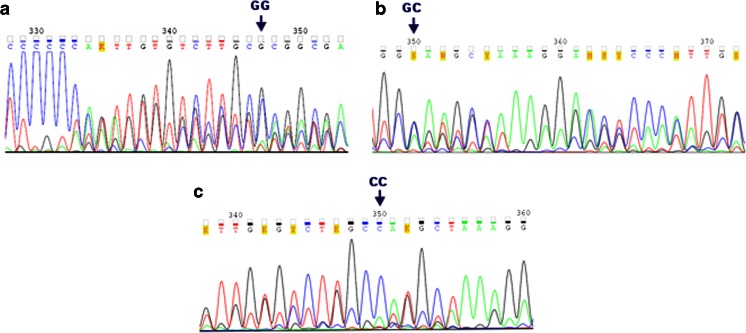
Chromatograms of DNA sequences, comprising *IL6* −174 G>C SNP (**a**, **b**, **c**). DNA forward strands were sequenced for all the analyzed SNPs. *GG*, *GC*, or *CC* genotypes at the described *IL6* SNP

*IL1A* −889 C>T, *IL1B* +3954 C>T, and *IL6* −174 G>C SNPs were genotyped, using self-designed nested polymerase chain reaction (PCR) assays. The used external and internal primer sequences, amplicon lengths, and annealing temperatures are shown in Table 1. External primer sequences were developed using the Vector NTI Suite 5.5 software, while the internal primers were based on published data [14, 18–21]. The amplifications were performed with a HotStarTaq® Master Mix Kit (Qiagen, Hilden, Germany). The PCR conditions were as follows: an initial activation for 15 min at 95 °C and for 40 cycles of repeated denaturation at 94 °C for 30 s, annealing at temperatures designed for the used primer pairs (see Table 1), for 1 min, and extension at 72 °C for 2 min, and a final extension at 72 °C for 10 min. The nested PCR products were resolved on 1 % agarose gels, stained with ethidium bromide, and then digested overnight with NcoI, TaqI, and Hsp92II endonucleases, used to determine genotypes at *IL1A* −889 C>T, *IL1B* +3954 C>T, and *IL6* −174 G>C SNPs, respectively (see Table 2). The products of digestion reactions were resolved on 2 % agarose gels. Genotypes were determined at the analyzed polymorphic sites by the length of restriction fragments, as described previously ([14, 18–21]; see Table 2, Fig. 1). The randomly selected PCR products, including the analyzed SNPs, were then verified by sequencing according to the Sanger method. In case of *IL1A* and *IL1B* SNPs, the sequencing was performed for seven and nine CC homozygotes, four and five CT heterozygotes, as well as for three and one TT homozygotes, respectively. Regarding the *IL6* SNP, sequencing was done for five GG homozygotes, five GC heterozygotes, and 12 CC homozygotes. Examples of chromatograms with DNA sequences of distinct genotypes at the studied polymorphisms are shown in Figs. 2 and 3. The sequenced and referenced DNA regions were compared using the BLASTN program for alignment of two (or more) sequences. The chromatograms were read out using the Sequence Scanner 1.0 and Chromas Lite 2.1.1 softwares.

**Table 1:** Primer sequences, annealing temperatures, and amplicon lengths obtained in the nested polymerase chain reaction (PCR) assays for *IL1A*, *IL1B*, and *IL6* polymorphisms

Gene	GenBank accession no.	SNP name	Primer sequences (5′–3′)		Annealing temperature (°C)	Amplicon length (bps)
*IL1A*	NC_000002	−889 C>T (rs1800587)	External	For: AAACAGGAACAGAGGGAATACTT	52	485
				Rev: CTGTAGAAGAAGGTGTGTGCAAG		
			Internal	For: GGGGGCTTCACTATGTTGCCCACACTGGACTAA	57	309
				Rev: GAAGGCATGGATTTTTACATATGACCTTCCATG		
*IL1B*	NC_000002	+3954 C>T (rs1143634)	External	For: GTAATAGACCTGAAGCTGGAACC	52	383
				Rev: CCATTTACCTTGTTGCTCCATA		
			Internal	For: GTTGTCATCAGACTTTGACC	59	249
				Rev: TTCAGTTCATATGGACCAGA		
*IL6*	NC_000007	−174 G>C (rs1800795)	External	For: GAAGTAACTGCACGAAATTTGAG	52	543
				Rev: GATAAATCTTTGTTGGAGGGTGA		
			Internal	For: CAGAAGAACTCAGATGACTG	58	431
				Rev: GTGGGGCTGATTGGAAACC		

*No.* number; *SNP* single nucleotide polymorphism; *bps* base pairs

**Table 2:** Length of restriction fragments and genotypic profiles at *IL1* and *IL6* polymorphisms

*IL* SNP	Restriction enzyme	Profile (bps)
*IL1A* −889 C>T	NcoI	CC: 266, 43
		CT: 309, 266, 43
		TT: 309
*IL1B* +3954 C>T	TaqI	CC: 135, 114
		CT: 249, 135, 114
		TT: 249
*IL6* −174 G>C	Hsp92II	GG: 229, 173, 29
		GC: 229, 173, 122, 51, 29
		CC: 229, 122, 51, 29

*SNP* single nucleotide polymorphism; *bps* base pairs

### Statistical analysis

The prevalence rates of genotypes and alleles at the *IL1* and *IL6* polymorphisms in the infected and uninfected fetuses and neonates were estimated by means of descriptive statistics. The analyzed groups of patients were studied for the Hardy–Weinberg (H-W) equilibrium, the linkage disequilibrium (LD), and haplotypes, using the SNPStats software (http://bioinfo.iconcologia.net/en/SNPStats_web). The correlation of the genotypes, alleles, or haplotypes at *IL* SNPs with the development of congenital *T. gondii* infections was determined by cross-tabulation, Pearson’s Chi-squared, as well as by the logistic regression model. The analysis of haplotypes at *IL1* SNPs and the multiple-SNP analysis were carried out by the expectation–maximization (EM) algorithm. The study results were estimated as being statistically significant when the significance level was *p* ≤ 0.050. The statistical analysis was, in part, supported by the NCSS 97 software.

## Results

### Hardy–Weinberg equilibrium, linkage disequilibrium

The genotypes at all the analyzed polymorphic sites preserved the H-W equilibrium in the fetuses and neonates infected with *T. gondii* (*p* = 0.081, *p* = 0.430, and *p* = 0.680, for *IL1A* −889 C>T, *IL1B* +3954 C>T, and *IL6* −174 G>C SNPs, respectively). Taking into account the control cases, the genotypes at *IL1B* +3954 C>T were in H-W equilibrium (*p* = 0.150), while the variants at *IL1A* −889 C>T and *IL6* −174 G>C were not (*p* ≤ 0.050). *IL1A* −889 C>T and *IL1B* +3954 C>T SNPs were seen in LD between the infected and the control groups of patients (*p* ≤ 0.050).

### Prevalence rates of the genotypes at *IL1A*, *IL1B*, and *IL6* SNPs

In the offspring infected with *T. gondii*, the prevalence rates of CC, CT, and TT genotypes at *IL1A* −889 C>T/*IL1B* C>T SNPs were 63.6 % (14/22)/72.7 % (16/22), 22.7 % (5/22)/22.7 % (5/22), and 13.6 % (3/22)/4.5 % (1/22), respectively (see Table 3). Among the control cases, the prevalence rates of the analyzed variants were 51.0 % (25/49)/57.1 % (28/49), 26.5 % (13/49)/30.6 % (15/49), and 22.4 % (11/49)/12.2 % (6/49), respectively. Taking into account the *IL6* −174 G>C SNP, GG, GC, and CC genotypes were carried by 27.3 % (6/22), 45.5 % (10/22), and 27.3 % (6/22) of the infected offspring, respectively. In control cases, the prevalence rates of GG, GC, and CC variants at the *IL6* SNP were 57.1 % (28/49), 22.4 % (11/49), and 20.4 % (10/49), respectively. Considering the *IL6* SNP, the GC heterozygotes were significantly associated with toxoplasmosis and increased the risk of *T. gondii* infection [odds ratio (OR) 4.24, 95 % confidence interval (CI) 1.24–14.50 in the codominant model, and OR 3.56, 95 % CI 1.19–10.64 for GC and CC genotypes in the dominant model; *p* ≤ 0.050; see Table 3]. Regarding the *IL1A* and *IL1B* SNPs, the observed genotypes were distributed similarly between the infected and uninfected offspring. Neither the haplotype analysis performed for *IL1* SNPs nor the multiple-SNP analysis for all the analyzed polymorphisms showed any differences in the prevalence rates of distinct variants between the studied groups.

**Table 3:** Single-SNP analysis of the relationship between genotypes at the *IL1* and *IL6* polymorphisms and the occurrence of congenital *Toxoplasma gondii* infection

Gene polymorphism	Genetic model	Genotype	Genotype prevalence rates, *n* (%)		OR (95 % CI)	*p*-Value^a^
			Infected cases	Controls		
*IL1A* −889 C>T	Codominant	CC	14 (63.6 %)	25 (51.0 %)	1.00	0.560
		CT	5 (22.7 %)	13 (26.5 %)	0.69 (0.20–2.33)	
		TT	3 (13.6 %)	11 (22.4 %)	0.49 (0.12–2.04)	
	Dominant	CC	14 (63.6 %)	25 (51.0 %)	1.00	0.320
		CT-TT	8 (36.4 %)	24 (49.0 %)	0.60 (0.21–1.67)	
	Recessive	CC-CT	19 (86.4 %)	38 (77.5 %)	1.00	0.380
		TT	3 (13.6 %)	11 (22.4 %)	0.55 (0.14–2.19)	
	Overdominant	CC-TT	17 (77.3 %)	36 (73.5 %)	1.00	0.730
		CT	5 (22.7 %)	13 (26.5 %)	0.81 (0.25–2.66)	
*IL1B* +3954 C>T	Codominant	CC	16 (72.7 %)	28 (57.1 %)	1.00	0.370
		CT	5 (22.7 %)	15 (30.6 %)	0.58 (0.18–1.91)	
		TT	1 (4.5 %)	6 (12.2 %)	0.29 (0.03–2.64)	
	Dominant	CC	16 (72.7 %)	28 (57.1 %)	1.00	0.200
		CT-TT	6 (27.3 %)	21 (42.9 %)	0.50 (0.17–1.50)	
	Recessive	CC-CT	21 (95.5 %)	43 (87.8 %)	1.00	0.280
		TT	1 (4.5 %)	6 (12.2 %)	0.34 (0.04–3.02)	
	Overdominant	CC-TT	17 (77.3 %)	34 (69.4 %)	1.00	0.490
		CT	5 (22.7 %)	15 (30.6 %)	0.67 (0.21–2.14)	
*IL6* −174 G>C	Codominant	GG	6 (27.3 %)	28 (57.1 %)	1.00	≤0.050
		GC	10 (45.5 %)	11 (22.4 %)	4.24 (1.24–14.50)	
		CC	6 (27.3 %)	10 (20.4 %)	2.80 (0.73–10.72)	
	Dominant	GG	6 (27.3 %)	28 (57.1 %)	1.00	≤0.050
		GC-CC	16 (72.7 %)	21 (42.9 %)	3.56 (1.19–10.64)	
	Recessive	GG-GC	16 (72.7 %)	39 (79.6 %)	1.00	0.530
		CC	6 (27.3 %)	10 (20.4 %)	1.46 (0.46–4.70)	
	Overdominant	GG-CC	12 (54.5 %)	38 (77.5 %)	1.00	0.054
		GC	10 (45.5 %)	11 (22.4 %)	2.88 (0.98–8.43)	

*p* ≤ 0.050 is considered significant

*n* number of tested fetuses and newborns; *OR* odds ratio; *CI* confidence interval

^a^Logistic regression model

### Distribution of the alleles at *IL1* and *IL6* SNPs

Among the infected fetuses and neonates, the prevalence rates of C and T alleles at *IL1A*/*IL1B* SNPs were 75.0 % (33/44)/84.1 % (37/44) and 25.0 % (11/44)/15.9 % (7/44), respectively (see Table 4). In case of the uninfected patients, the analyzed alleles were found in 64.3 % (63/98)/72.4 % (71/98) and 35.7 % (35/98)/27.6 % (27/98) of the offspring, respectively. Taking into account the *IL6* SNP, the G and C alleles were identified in 50 % (22/44) of the infected cases for each allele. In the uninfected controls, the prevalence rates of G and C alleles were 68.4 % (67/98) and 31.6 % (31/98), respectively. A cross-tabulation analysis showed that alleles at the *IL1A* polymorphic site were similarly distributed between *T. gondii*-infected and -uninfected fetuses and neonates (see Table 4). In case of the *IL1B* SNP, the major C allele was significantly more frequent among the infected than among the uninfected offspring (χ^2^ = 7, *p* ≤ 0.050; Fisher’s exact test). Considering the *IL6* SNP, the minor C allele was significantly more frequent among the infected versus the uninfected cases (χ^2^ = 4, *p* ≤ 0.050; Fisher’s exact test).

**Table 4:** Distribution of the alleles located at the *IL1* and *IL6* SNPs

Gene polymorphisms and alleles		No. of carriers with *IL* alleles (%)		*p*-Value
		Infected cases	Controls	
*IL1A* −889 C>T	C	33 (75.0)	63 (64.3)	0.207^a^
	T	11 (25.0)	35 (35.7)	
*IL1B* +3954 C>T	C	37 (84.1)	71 (72.4)	≤0.050^b^
	T	7 (15.9)	27 (27.6)	
*IL6* −174 G>C	G	22 (50.0 %)	67 (68.4 %)	≤0.050^b^
	C	22 (50.0 %)	31 (31.6 %)	

*p* ≤ 0.050 is considered significant

*No.* number

^a^Pearson’s Chi-squared test

^b^Fisher’s exact test

## Discussion

In our study, we found that infants and neonates with GC genotypes at the *IL6* −174 G>C SNP were at an approximately four times higher risk of congenital infection with *T. gondii* as compared to GG and CC homozygotes. Moreover, the minor C allele at the studied locus was significantly more prevalent among the infected than in the uninfected offspring. Regarding *T. gondii* infections, only one previous study, performed for patients with TR and control cases, reported the role of the *IL6* −174 G>C polymorphism for the occurrence of disease [14]. Similarly to our results, the prevalence rates of distinct genotypes at the analyzed *IL6* locus were significantly different between the study groups of patients and control cases. In patients with TR, the GC heterozygotic status at the SNP was estimated to be correlated to occurrence of the disease as well [14]. The significantly higher prevalence rate of the C allele versus the G allele observed in our study among the *T. gondii*-infected offspring as compared to the control cases was also estimated for TR patients [14]. Previously, the *IL6* −174 G>C polymorphism was determined as being associated with altered expression levels of the encoded cytokine as well [22–24]. Studies on the role of the *IL6* −174 G>C SNP in RSV-infected macrophages showed GC heterozygotes and CC homozygotes as high IL6 production genotypes, correlated with the infection, while among RSV-infected patients, the CC genotype at the analyzed SNP was associated with low IL6 levels [22, 23]. Taking into account the papers reporting increased IL6 levels to be correlated with *T. gondii* infection in various patient groups, it seems possible that the GC heterozygotic status at the *IL6* −174 G>C SNP may alter the immune response against the parasite through higher IL6 cytokine production. However, a detailed description of IL6 molecular changes, possibly associated with congenital toxoplasmosis development, would be a challenge. So far, the C allele at the *IL6* −174G>C SNP was reported to generate new binding sites for NF-1 and Smad4 transcription factors, which are not observed in the presence of the G allele [25, 26]. In addition, a variable AnTn tract, located at the nucleotides from −392 up to −373 within the *IL6* gene promoter, was also reported to affect the transcription of −174 G allele and −174 C allele-related haplotypes [27].

According to genetic modifications localized in *IL1* genes, we found the major C allele at the *IL1B* +3954 C>T SNP to be significantly more frequent among the fetuses and neonates with congenital *T. gondii* infection than among the uninfected cases. So far, several studies showed some involvement of the proinflammatory IL1β cytokine in an immune response after *T. gondii* infection, although the results are ambiguous [8, 11, 28]. Taking into account *IL1* polymorphisms, a previous study in TR patients showed, likewise to our outcomes, similar distributions of the genotypes and, in addition, of the alleles at the analyzed *IL1A* −889 C>T and *IL1B* +3954 C>T SNPs in patients with TR and in control cases [15]. The outcomes obtained in our study may suggest that the presence of mutated T allele at the analyzed *IL1B* SNP plays a protective function against the development of congenital toxoplasmosis. However, the mechanism of the role of the *IL1B* 3954 C>T SNP should be investigated in a detailed molecular study.

Considering our outcomes, as presented in this paper, the GC heterozygotic status at the *IL6* −174 G>C SNP, as well as the presence of C alleles at both *IL6* and *IL1B* +3954 C>T SNPs, seems to have been involved in the occurrence of congenital toxoplasmosis in the studied fetuses and neonates. So far, several studies have shown some contribution of the encoded IL6 and IL1β cytokines to immune responses against *T. gondii*, although no such research has been performed for congenital toxoplasmosis. In case of *IL6*, the GC heterozygotic status at the −174 G>C SNP was determined to be correlated with TR development, while no such relationship was observed for either the *IL1A* −889 C>T or *IL1B* +3954 C>T SNPs. Based on our outcomes, we suggest a possible contribution of GC heterozygotic status at the *IL6* −174 G>C SNP to the immune response efficacy against congenital *T. gondii* infection via an increased synthesis of the IL6 cytokine, whereas the T allele at the *IL1B* +3954 C>T SNP might be protective against the infection. Although the character of observed genetic changes is suggestive of their effective relationship with congenital toxoplasmosis, a detailed further mechanistic research should be undertaken to investigate the hypothesis in detail. We also suggest that further studies on the role of *IL6* and *IL1* SNPs, performed with larger groups of patients with congenital toxoplasmosis, would be beneficial to verify our results.
